# In Situ Raman Spectroscopy as a Valuable Tool for Monitoring Crystallization Kinetics in Molecular Glasses

**DOI:** 10.3390/molecules29194769

**Published:** 2024-10-09

**Authors:** Roman Svoboda, Nicola Koutná, Magdalena Hynková, Marek Pakosta

**Affiliations:** 1 Department of Physical Chemistry, Faculty of Chemical Technology, University of Pardubice, nam. Cs. legii 565, 532 10 Pardubice, Czech Republic; 2 Faculty of Electrical Engineering and Informatics, University of Pardubice, nam. Cs. legii 565, 530 02 Pardubice, Czech Republic

**Keywords:** in situ Raman microscopy, DSC, crystal growth kinetics, amorphous drugs

## Abstract

The performance of in situ Raman microscopy (IRM) in monitoring the crystallization kinetics of amorphous drugs (griseofulvin and indomethacin) was evaluated using a comparison with the data obtained via differential scanning calorimetry (DSC). IRM was found to accurately and sensitively detect the initial stages of the crystal growth processes, including the rapid glass–crystal surface growth or recrystallization between polymorphic phases, with the reliable localized identification of the particular polymorphs being the main advantage of IRM over DSC. However, from the quantitative point of view, the reproducibility of the IRM measurements was found to be potentially significantly hindered due to inaccurate temperature recording and calibration, variability in the Raman spectra corresponding to the fully amorphous and crystalline phases, and an overly limited number of spectra possible to collect during acceptable experimental timescales because of the applied heating rates. Since theoretical simulations showed that, from the kinetics point of view, the constant density of collected data points per kinetic effect results in the smallest distortions, only the employment of the fast Raman mapping functions could advance the performance of IRM above that of calorimetric measurements.

## 1. Introduction

Raman spectroscopy (RS) represents a key experimental technique for the pharmaceutical industry, research, and development. As a highly versatile method, applicable to solids, liquids (even aqueous solutions, without any interference from H_2_O), and gasses, RS can be employed in practically all stages of pharmaceutical production; RS is particularly useful for distinguishing between different polymorphic forms of a compound, which is crucial in ensuring the efficacy and stability of the final drug product. With high chemical specificity (due to the ability to detect distinct molecular vibrations), RS can reliably identify active pharmaceutical ingredients (APIs) and impurities with great accuracy even in complex mixtures. Since RS is a non-destructive technique with little to no sample preparation requirements, its utilization can be well automated, even for expensive or sensitive materials [1,2,3,4,5].

A further major improvement of the RS technique is achieved via the integration of optical microscopes, which enable the spatial and depth (confocal) mapping of a sample—with the resolution typically being ~1–3 μm [6,7,8]. Probably the most important RS accessory is, however, a temperature stage/table, which allows for the continuous collection of the Raman spectra during various temperature programs (usually linear heating, linear cooling, or isothermal annealing). This experimental setup, denoted as in situ Raman spectroscopy (IRS) or in situ Raman microscopy (IRM) paired with a fast mapping option (commonly realized via a multichannel detector), represents the latest advancement utilized as a high-end option in pharmaceutical research and quality control. IRM not only enables the real-time monitoring of chemical reactions and processes but also does so under practically any desired experimental conditions since the temperature stages (as well as the microscope’s optical elements that secure the path for the laser beam and Raman emission) can operate under a wide range of temperatures, pressures, and atmospheres [9,10,11].

Since one of the latest trends in pharmaceutical research involves the extensive utilization of amorphous APIs (with the main benefits being significantly increased water solubility and, consequently, the bioavailability of a drug [12,13,14,15]), IRM has also been naturally employed in monitoring the degradation of the amorphous phase under various conditions [16,17,18,19,20,21,22,23,24,25]. In this regard, the spontaneous recrystallization of the amorphous phase (induced via, e.g., an increased temperature, contact with moisture, or certain types of irradiation) is the most common and important degradation process—the formation of the crystalline phase within the amorphous API matrix could significantly decrease the bioavailability of the drug, effectively decreasing the dosage given to the patient and leading to harm or at least an ineffective treatment. Whereas IRM is readily used to study the crystallization behavior of amorphous APIs [26,27,28,29,30], it is only very rarely utilized to its full potential, which would be an imitation of standard calorimetric crystallization kinetic studies (commonly performed by means of differential scanning calorimetry, DSC). This is rather surprising, as IRM not only provides information about the amorphous→crystalline transition (akin to the DSC data) but also, at the same time, the potential simultaneous/overlapping formation of different API polymorphs is clearly revealed, monitored, and quantified [31].

In the present paper, a direct comparison between the IRM and DSC data obtained under identical conditions for two selected APIs will be introduced. In the case of amorphous griseofulvin (GRIS, an anti-fungal drug with anti-viral and anti-cancer properties), a simple amorphous-to-crystalline transformation was studied, with no polymorphic changes involved. On the other hand, in the case of amorphous indomethacin (IMC, a non-steroidal anti-inflammatory drug), a competition between two polymorphs was recorded by means of both experimental techniques (IRM and DSC). The main goal of the present paper is to consider the suitability of the IRM data for advanced kinetic calculations and predictions, similar to those obtainable based on the DSC data. The specificity, benefits, and flaws of the two experimental techniques will be discussed for the two types of crystallization data; a simple guide for achieving maximum efficiency and accuracy during the IRM crystallization kinetics measurements was compiled. In addition to the experimental assessment of the IRM-based crystallization kinetics evaluation, a section devoted to the theory of step-based kinetics calculations (akin to what was published for the in situ X-ray measurements in [32]) will introduce the theoretically simulated data, demonstrating the potential deviations/distortions caused by the cumulative nature of IRM spectra recording.

## 2. Results

This section is divided into two sub-sections, introducing the results for the two respective materials—amorphous 20–50 μm powders of GRIS and IMC. Note that this specific particle size fraction was selected due to the relative ease of preparation (not needing overly rough grinding and potentially causing the partial formation of the crystalline phase), together with the certainty of a large amount of mechanical defects being present on each grain of powdered material. Low-molecular glasses, such as IMC and GRIS, crystallize dominantly from surface mechanical defects (e.g., micro-cracks, edges, or tips) [33,34,35,36,37]. In each sub-section, the raw DSC and IRM data will be introduced together with their base interpretation, focusing on the types of monitored crystal growth specific to the given API. A direct comparison between the individual growth stages, as monitored using the respective experimental techniques, will be emphasized. Note that the discussion of these results in terms of crystal growth kinetics will be introduced later, in Section 3.

### 2.1. Griseofulvin

**Figure 1:**
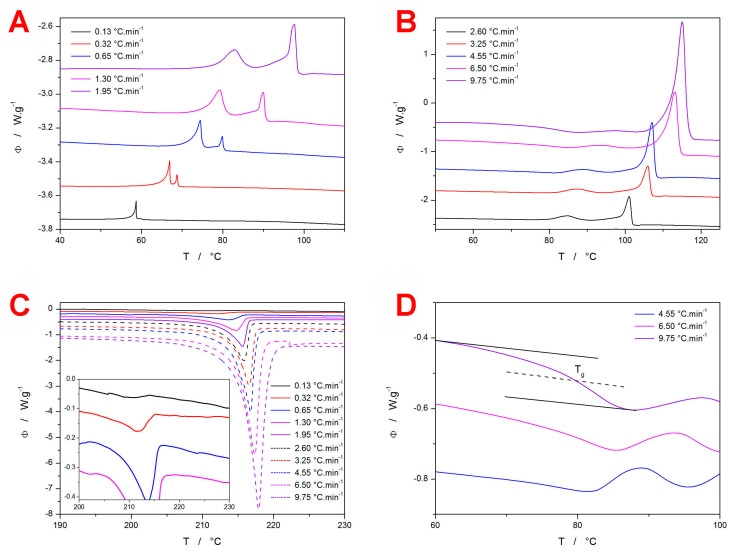
(**A**) DSC crystallization peaks obtained at a low q^+^ for the 20–50 µm GRIS powder. (**B**) DSC crystallization peaks obtained at a high *q*^+^ for the 20–50 µm GRIS powder. (**C**) DSC melting peaks obtained for the 20–50 µm GRIS powder; the inset shows a zooming-in on the curves measured at a low *q*^+^. (**D**) Selected DSC curves zoomed in on the glass transition region; an evaluation of the half-height midpoint *T_g_* is indicated. The solid black lines represent extrapolated temperature dependences of heat flow in the glassy and undercooled liquid regions; the dashed black line indicates a half distance between the black solid lines.

The calorimetric data obtained at different heating rates, *q*^+^, for amorphous GRIS powder (20–50 μm) are shown in Figure 1. Given the aim of the present study, the crystallization signals were, naturally, the main interest—see Figure 1A,B. It is immediately apparent that, at the highest *q*^+^, the weak glass transition signal (an endothermic step-like change in the heat capacity at ~80 °C) was followed by a practically imperceptible exothermic crystallization pre-peak (at ~97 °C) and then by the main exothermic crystallization peak between 105 and 115 °C. With a decreasing *q*^+^, the crystallization pre-peak increased in intensity (at the expense of the main crystallization peak), and it started to overlap with the glass transition effect. At slower heating than approximately 3 °C·min^−1^, even the onset of the glass transition effect disappeared under the exothermic crystallization pre-peak, which unambiguously indicates that this crystallization signal corresponds to the surface crystal growth, for which molecular self-diffusion is not limited by the frozen-in glassy state (associated with very large viscosities, *η* > 10^12^ Pa·s) that exist in the bulk material below *T_g_*. The occurrence of the crystal growth below *T_g_* can be, in the case of low-molecular glasses, associated with a rapid increase in the growth rate [33,34,35,36,37] (so-called glass–crystal GC growth) due to the surface mobility not being disrupted/hindered because of the re-organization of the amorphous phase (which is relatively quick and intense at *T* > *T_g_*). The existence and dominance of GC growth in amorphous GRIS is well documented with the increasing proportional intensity of the crystallization pre-peak at a lower *T*; the pre-peak and main peak become roughly equivalent in their magnitudes at *q*^+^ ≈ 1.5 °C·min^−1^, and with the decreasing *q*^+^, this ratio further increases in favor of the pre-peak (corresponding to GC growth).

Kinetics-wise, both crystallization processes exhibit a marked change in their shape/asymmetry with the *q*^+^—see Figure 1A,B. The GC growth DSC peak exhibits, at the lowest *q*^+^, a typically gradual increase in intensity, with the peak being of marked negative asymmetry (a slow onset and a sharp endset edge), which is characteristic of zero-order kinetics. The corresponding physico-chemical interpretation stems from a relatively compact surface crystalline layer slowly propagating inwards, through the volume of the amorphous grain; this is in perfect agreement with the large difference in the surface and bulk self-diffusion coefficients, where the surface is completely covered by the crystalline layer via the lateral crystal growth before significant propagation via the slower vertical growth starts (akin to, e.g., [38,39,40]). With an increasing *q*^+^, the asymmetry of the crystallization pre-peak gradually changes toward the first-order kinetics, which indicates the standard nucleation-growth process and the growth originating from a lower population of the energetically favored centers (i.e., micro-cracks, mechanical defects, etc.). Similar development of the peak shape also occurs for the main crystallization peak at the lowest *q*^+^, but it is very soon (*q*^+^ ≥ 0.5 °C·min^−1^) replaced with a complex exothermic crystallization signal. The complexity manifests as a consequent reaction/transformation mechanism consisting of kinetically decelerated crystal growth that occurs as a direct consequence (i.e., continuation) of the GC growth (possibly preferentially along the volume micro-cracks and defects), and a subsequent nucleation-growth sub-process (very probably initiated via secondary nucleation on the previously formed crystallites). With an increasing T (achieved via ↑ *q*^+^), the standard above *T_g_* growth becomes dominant due to the lack of the GC growth-formed crystalline phase and/or the consequently directly propagated crystal aggregates.

By assessing the position and shape of the corresponding endothermic melting peaks (see Figure 1C), it can be concluded that the dominant polymorphic form I is formed during all above-described crystal growth sub-processes in the amorphous GRIS powder (indicated by the extrapolated onset between 207 and 215 °C). The rather widespread melting peaks developing at *q*^+^ < 2 °C·min^−1^ indicate a wide distribution in quality (size, morphology, and location) for the crystallites formed within the particular growth sub-processes. In Figure 1D, the zoomed-in glass transition regions are shown for the highest *q*^+^; the determination of the half-height midpoint *T_g_* (≈80 °C) is demonstrated. The characteristic temperatures and enthalpies associated with the DSC data depicted in Figure 1 are summarized in Table 1.

**Figure 2:**
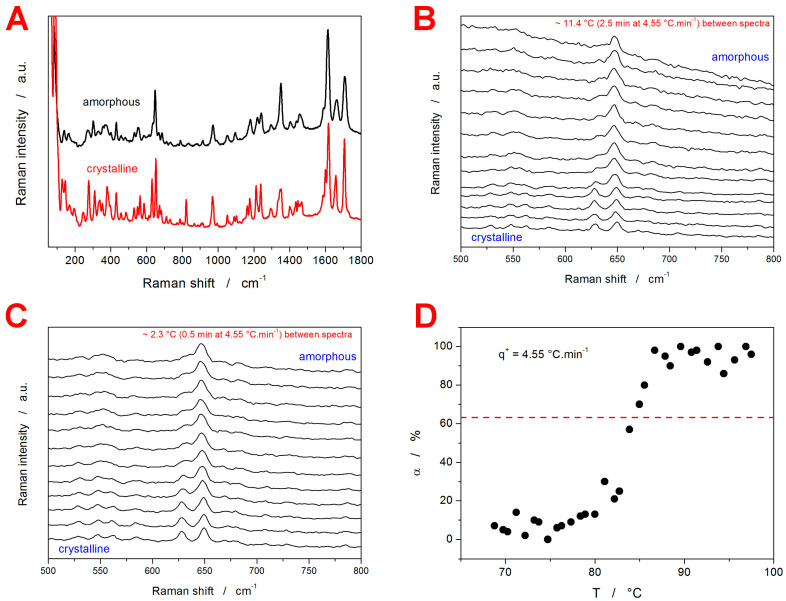
(**A**) Example Raman spectra of fully amorphous and fully crystalline GRIS. (**B**) Example series of Raman spectra monitoring the crystallization of the 20–50 µm GRIS powder at *q*^+^ = 4.55 °C·min^−1^. The spectra are zoomed in on the spectral region of interest; roughly every tenth spectrum is displayed for clarity. (**C**) Raman spectra similar to those from (**B**); fine selection of the spectra (roughly every second measured spectrum) collected at the time of the amorphous-to-crystalline transition is shown. (**D**) Temperature evolution of α determined by means of the multicomponent analysis from the data depicted in (**B**). The horizontal dashed line indicates α = 0.63, which was used to evaluate the characteristic temperature, *T_IRM_*.

During the in situ Raman microscopy (IRM) measurements (described later, in Section 4), the Raman signal gradually changed from the amorphous variant to the crystalline variant. The corresponding borderline spectra (fully amorphous and fully crystalline) are shown in Figure 2A. The following attribution of the Raman bands was suggested [41] for GSF: 0–200 cm^−1^ translational and vibrational lattice modes; 350–460 cm^−1^ and 590–690 cm^−1^ C–Cl vibration; 823 cm^−1^ cyclohexene ring; 968 and 1659 cm^−1^ benzene ring; 1150–1200 cm^−1^ C–O–C vibrations; 1200–1420 cm^−1^ CH, CH_2_, and CH_3_ deformations; and 1570–1635 and 1585–1735 cm^−1^ C=O vibrations. As one of the largest differences between the amorphous and crystalline GSF Raman spectra occurs in the 600–700 cm^−1^ spectral range (attributed to the C–Cl vibration), this region was chosen to assess the nature of the crystallization pre-peak. It should be noted that, for each API, several spectral regions (including the low-frequency part, which is very sensitive to the difference between the amorphous and crystalline phases [41]) were tested during the performance of the multicomponent analysis. The problem with the low-frequency data was the uncertainty of the associated background/baseline, which introduced significantly larger errors and irreproducibility to the evaluation of the degree of crystallinity. Hence, spectral regions with newly appearing bands and clearly identifiable baseline were selected for the α_c_ evaluation.

In Figure 2B,C, the examples of the development of the Raman spectra during the IRM experiment are shown. Figure 2B depicts the zoomed-in spectral region of interest (610–675 cm^−1^); roughly every tenth measured spectrum is shown for clarity. Figure 2C depicts a fine selection of the Raman spectra collected at the time of the amorphous-to-crystalline transition; roughly every second measured spectrum is shown for clarity. The full set of the measured spectra is shown in the Supplementary Materials online, together with the visually indicated portion of the signal used for the evaluation of the degree of crystallinity, α_c_ (using the multicomponent analysis in the Omnic Specta 2.1 software). Note that this specific region was selected as a part of the spectrum with the most pronounced value between the two borderline signals. Figure 2C then depicts an example of the α_c_-T dependence obtained for *q*^+^ = 4.55 °C·min^−1^, where each point corresponds to one processed spectrum from Figure 2B. Notably, the scatter in the data was caused by the following factors: (1) the slight ambiguity of the spectrum baseline subtraction; (2) the overall fluctuation of the Raman signal intensity (the physical spot from which the spectra are collected can become defocused due to the changes in the GRIS grain position—both the thermal expansion and surface corrugation associated with forming the crystalline phase can play their roles here); (3) several overlapping crystal growth mechanisms can manifest within the spot range of the Raman spectra collection, where each morphology can produce slightly different intensity of the Raman counts. The horizontal dashed line in Figure 2C indicates the fingerprint α_c_ value, which is characteristic of the nucleation-growth kinetics [42], and in the case of the validity of the nucleation-growth model [43,44,45,46], it should be close to the inflection point of the α_c_-*T* dependence (which can be considered fulfilled in the present case).

**Figure 3:**
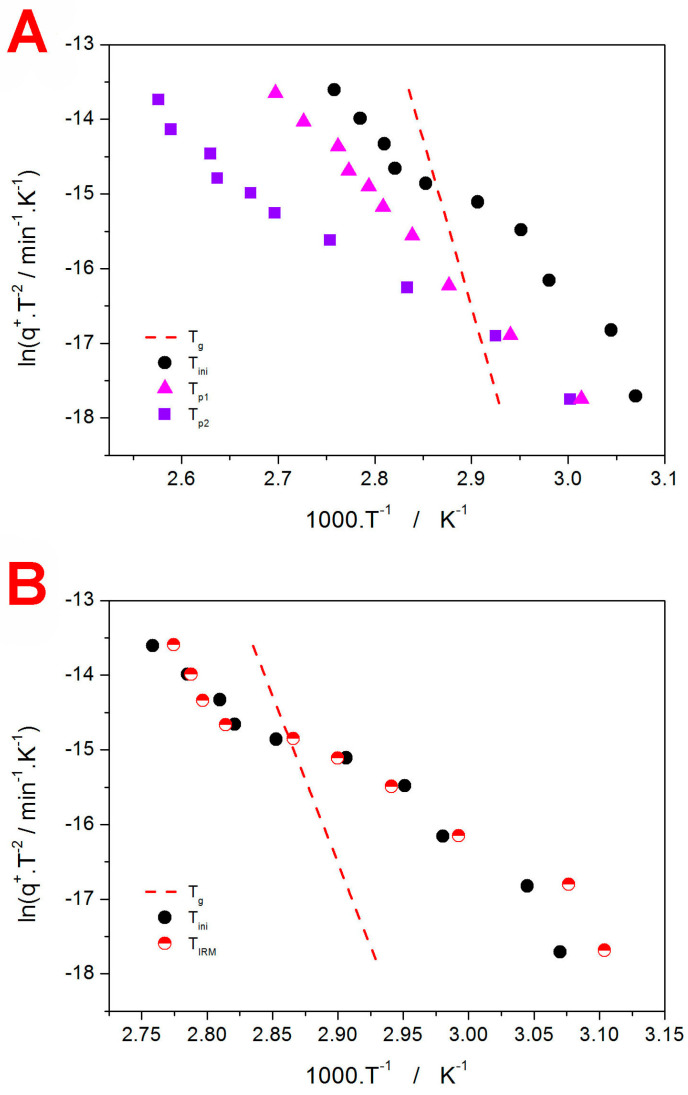
(**A**) Kissinger plot obtained for the DSC crystallization of the amorphous 20–50 µm GRIS powder—data shown in Figure 1A,B. The quantities *T_ini_*, *T_p_*_1_, and *T_p_*_2_ correspond to the temperatures of the initial crystallization onset, first crystallization peak, and second crystallization peak, respectively. The dashed line indicates the estimated development of *T_g_* with q^+^. (**B**) Kissinger plot comparing the GRIS dependencies obtained for *T_ini_*, *T_IRM_*, and *T_g_*.

One of the best ways to compare the *q*^+^-dependent crystallization behavior [42] is by means of so-called Kissinger plots [47]—see Figure 3. For the standard single-process kinetics with Arrhenian-type activation energy [48] (which is common for the crystallization processes from the amorphous phase), the Kissinger plot is a linear dependence. In Figure 3A, the DSC data for the different stages of the crystal growth processes are transformed into the Kissinger dependencies: *T_ini_* stands for the onset temperature of the crystallization pre-peak (the GC growth process), *T_p_*_1_ stands for the temperature of the maximum of the crystallization pre-peak (the GC growth process), *T_p_*_2_ stands for the temperature of the maximum of the main crystallization peak. In addition, a pseudo-Kissinger dependence was also calculated for *T_g_* to indicate the mutual position of the crystal growth and glass transition processes—see the dashed line in Figure 3A. This calculation was based on the half-height midpoint *T_g_* value determined from the *q*^+^ = 9.75 °C·min^−1^ curve (see Figure 1D) and the value of the activation energy for the structural relaxation process Δh^*^ = 379 kJ·mol^−1^ [49]. The exact nature of the calculation was based on the novel accurate and reliable method for the determination of Δh^*^, which was also used in [49]. Note that the Kissinger transformation is an approximation utilized just as a mathematical operation that plots the crystallization and relaxation data into the same plot; the experimental evaluation of Δh^*^ still needs a specialized temperature program [49] (due to the memory effect of the relaxation kinetics [50,51,52]) inconsistent with the present simple heating scans used for studying the crystal growth processes. Whereas the *T_p_*-based dependencies exhibit acceptable linearity to be considered consistent single-process transformation mechanisms (at least activation energy-wise), the onset of the crystallization pre-peak exhibits a clear and marked curvature of the Kissinger dependence, the initiation of which is consistent with the growth starting to occur below *T_g_*. In particular, the Kissinger dependence is shifted to a lower *T*, indicating an earlier DSC-detectable initiation of the crystal growth, i.e., confirming that the crystal growth is markedly faster below *T_g_* (thus verifying its “GC” nature). Importantly, the indication of the significantly more rapid GC growth appears to be effectively detectable only from the *T_ini_*.

In Figure 3B, the DSC-measured Kissinger dependence for *T_ini_* is compared with the data obtained by means of IRM, with the characteristic temperatures evaluated in correspondence to α_c_ = 63% (see Figure 2B for an example). Clearly, in the case of the fine GRIS powder, *T_ini_* determined calorimetrically corresponds very well to the highest crystal growth rate detected via IRM (it needs to be borne in mind that IRM is a surface-monitoring technique, and for the low-molecular glasses, the GC crystal growth is always initiated from the surface mechanical defects). The apparent deviation at the lowest *q*^+^ may be (on the DSC side) a consequence of either a lower DSC detection limit (IRM seems to detect the manifestation of the GC growth even earlier than DSC) or of a possible inaccuracy of the DSC baseline subtraction procedure. From the IRM point of view, the possible factors contributing to this deviation might be the inaccuracy of the temperature calibration of the hot stage used for the Raman measurements, or the inequality of the states used to determine the characteristic temperatures *T_ini_* and *T_IRM_*. However, most importantly, the IRM technique appears to be equally adequate for detecting the GC crystal growth.

### 2.2. Indomethacin

**Figure 4:**
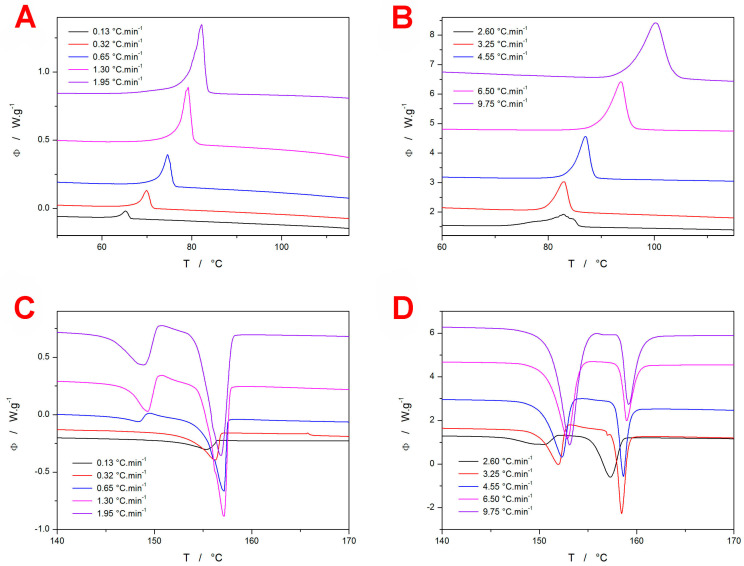
(**A**) DSC crystallization peaks obtained at a low *q*^+^ for the 20–50 µm IMC powder. (**B**) DSC crystallization peaks obtained at a high *q*^+^ for the 20–50 µm IMC powder. (**C**) DSC melting peaks obtained at a low *q*^+^ for the 20–50 µm IMC powder. (**D**) DSC melting peaks obtained at a high *q*^+^ for the 20–50 µm IMC powder.

The present section, dealing with the DSC and IRM results obtained for the crystallization of the amorphous IMC powder, will be structured similarly as the previous Section 3.1. In Figure 4, the zoomed-in exothermic DSC crystallization peaks (Figure 4A,B) and the endothermic DSC melting peaks (Figure 4C,D) are shown; the characteristic temperatures and enthalpies evaluated from these DSC data are listed in Table 2. The crystallization seemingly proceeds via a single-process transformation mechanism; one recorded exception was the measurement at 2.6 °C·min^−1^, which exhibited a clearly complex double-peak behavior due to the unsuitable experimental conditions (above-average temperature in the laboratory and prolonged pre-measurement processing time; a repeated measurement already exhibited a standard single-process signal). Asymmetry-wise, the development of the shape of the kinetic peaks is much less pronounced than in the case of GRIS; nonetheless, similar trends can also be observed for IMC: at a low *q*^+^, the peaks’ onsets are slow and prolonged, whereas at a higher *q*^+^, the asymmetry of the DSC peaks is strongly reminiscent of the nucleation-growth ones [42]. Interestingly, the single crystallization peak encompasses the formation of two IMC polymorphic phases, α (melting temperature *T_m_* ≈ 149 °C) and γ (*T_m_* ≈ 157 °C), as evidenced by the extrapolated onsets of the melting peaks from Figure 4C,D. As the α polymorph is the metastable one, it is preferentially formed under heightened mobility conditions (higher *T* or *q*^+^), under the presence of mechanical defects (in fine powders), or after prolonged free-surface nucleation [53,54]. Correspondingly, practically no α phase was formed at the lowest *q*^+^ (0.13 and 0.32 °C·min^−1^) despite the crystallization being monitored for a very fine IMC powder.

**Figure 5:**
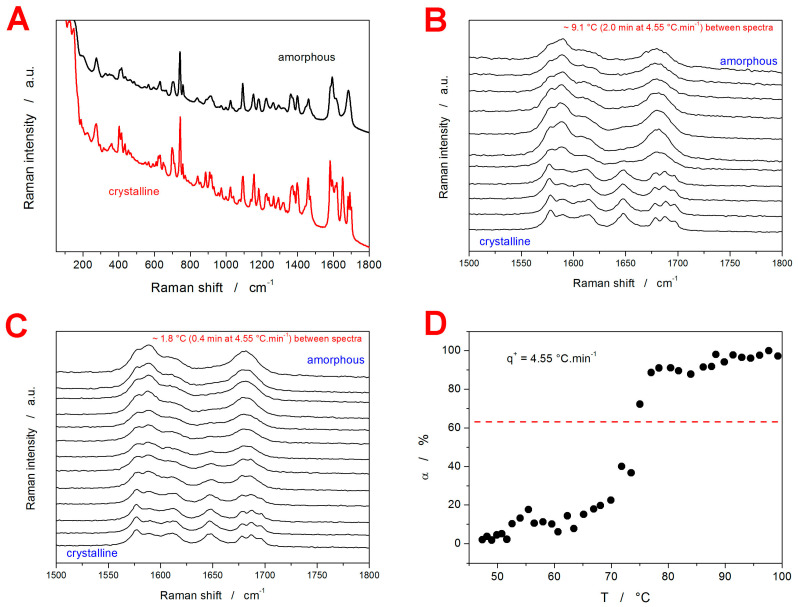
(**A**) Example Raman spectra of fully amorphous and fully crystalline IMC. (**B**) Example series of Raman spectra monitoring the crystallization of the 20–50 µm IMC powder at *q*^+^ = 4.55 °C·min^−1^. The spectra are zoomed in on the spectral region of interest; roughly every fifth spectrum is displayed for clarity. (**C**) Raman spectra similar to those from (**B**); fine selection of the spectra (every measured spectrum in that time frame) collected at the time of the amorphous-to-crystalline transition is shown. (**D**) Temperature evolution of α determined by means of the multicomponent analysis from the data depicted in (**B**). The horizontal dashed line indicates α = 0.63, which was used to evaluate the characteristic temperature *T_IRM_*.

The Raman spectra for fully amorphous and crystalline IMC powders are shown together with the examples of the IRM measurements performed at *q*^+^ = 4.55 °C·min^−1^, shown in Figure 5. The most important bands in the displayed Raman spectra can be assigned as follows: the amorphous IMC is characterized by the broad Raman band at 1685 cm^−1^, γ-IMC is characterized by the 1700 cm^−1^ band (benzoyl C=O stretching), and α-IMC is characterized by bands at 1650 (benzoyl C=O stretching), 1680 (benzoyl C=O stretching), and 1692 cm^−1^ (acid O–C=O stretching) [55,56].

A similar formal description as that in the case of GRIS (see Section 3.1) can also be used here. The monitored spectral range for IMC was ~1560–1720 cm^−1^. Technically, it would be possible to distinguish between the α and γ polymorphs in this spectral range. However, the absolute majority of the surface-located crystalline spots was found to be of the γ phase, and the occasional α phase signal was rather irreproducible when both found and developed (generally, the main reason was the very small spot from which the Raman spectra were collected). The occasional secondary occurrence of the α-phase signal within the crystalline IRM spectra can be an additional reason for the increased scatter of the α_c_-T data (as seen in Figure 5C); nevertheless, all the α_c_-*T* dependencies obtained at different *q*^+^ values for the IMC powder were of high enough quality for the T_IRM_ value to be accurately determined.

**Figure 6:**
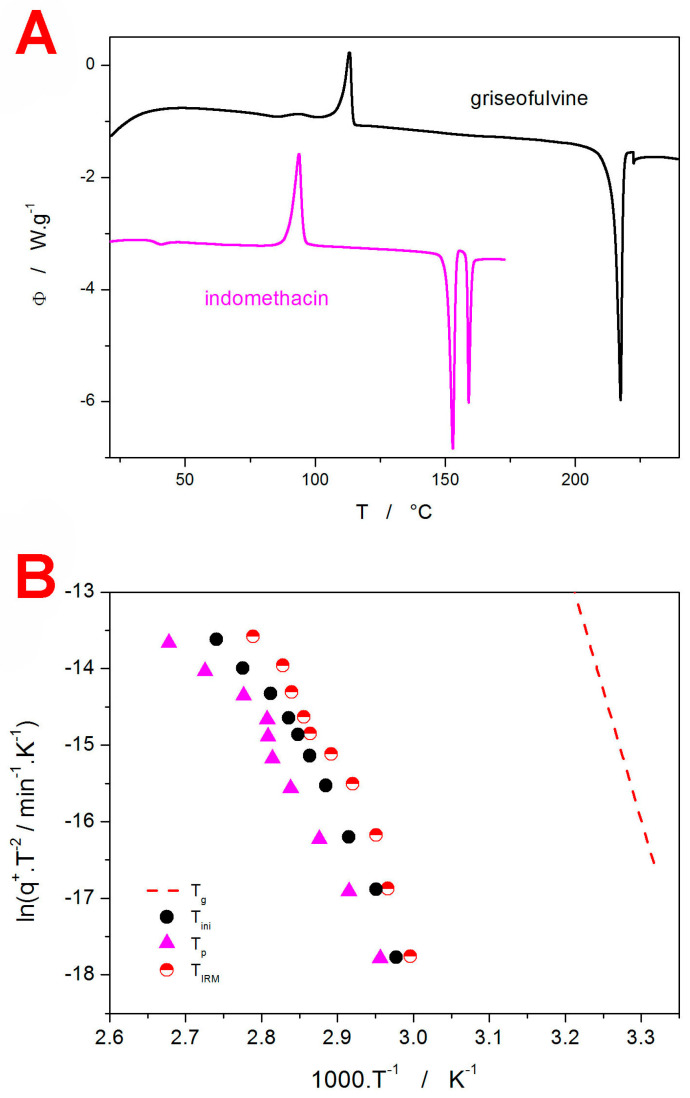
(**A**) Comparison of the DSC curves obtained for the GRIS and IMC powders at *q*^+^ = 6.50 °C·min^−1^. (**B**) Kissinger plot comparing the IMC dependencies obtained for *T_ini_*, *T_p_*, *T_IRM_*, and *T_g_*.

Before we compare the DSC and IRM data obtained for the IMC powder, note that the main difference between GRIS (discussed in Section 3.1) and IMC needs to be briefly reiterated. In the case of GRIS, the crystallization proceeds within (and below) the glass transition range, and it could thus be associated with rapid GC growth. The specificity of GRIS lies not only in its extremely high surface mobility [57] (compared to the other low-molecular organic glasses) but also in its ability to sustain the rapid GC growth mode at temperatures above *T_g_* (namely up to *T_g_* + 62 °C) [58]. Apart from providing clear evidence of the attribution of the GRIS crystallization pre-peak to the GC growth (the pre-peak is initiated below *T_g_*, but the GC growth continues well above *T_g_* as well, where, for most other low-molecular glasses, it would cease), the rapidity of the GRIS GC growth also represents the main difference between GRIS and IMC. The GC crystal growth in IMC does not seem to be detectable macroscopically via DSC, and the normal crystal growth occurs ~40 °C above *T_g_*—see Figure 6A for a comparison of the DSC curves obtained for the two low-molecular glasses at 6.50 °C·min^−1^. For that reason, the crystal growth cannot be influenced by the process of structural relaxation, as is also indicated in the corresponding Kissinger plot (see Figure 6B), where even the initial recording of the macroscopic crystallization *T_ini_* is well above *T_g_* and also well below *T_m_*. However, the Kissinger plots (monitored by both DSC and IRM) are significantly curved, which suggests potential *T*-dependent crystallization activation energy—this possibility will be, among others, discussed in the following section. It is also noteworthy that the IRM-detected peak of the surface crystal growth is slightly below (temperature-wise) the initial onset of the macroscopically monitored (via DSC) crystallization signal. This is in good agreement with the expected high surface sensitivity of the IRM technique.

## 3. Discussion

The present section is divided into two sub-sections. The first one will focus on advanced kinetic calculations (based mostly on the DSC data) describing the measured crystallization kinetics and an associated critical evaluation of the quality of the IRM data in this regard. The second section will assess the IRM technique from a fundamental point of view (using theoretically simulated data), discussing its limitations with regard to the non-instantaneous collection of the measured signal.

### 3.1. Advanced Crystallization Kinetics

Calorimetrically studied crystallization kinetics (and, by extension, practically all related α-*T* and dα·d*t*^−1^-*T* dependencies) is, by default, described in terms of the Arrhenian solid-state kinetic equation [48]:

(1) $$\Phi =∆H\cdot A\cdot e^{-E/RT}\cdot f(\alpha_{c})$$

where *Φ* is the measured DSC heat flow, Δ*H* is the crystallization enthalpy, *A* is the pre-exponential term, *E* is the crystallization activation energy, *R* is the universal gas constant, *T* is temperature, and *f*(*α_c_*) stands for a mathematical function expressing the shape of the crystallization peak as a dependence of the degree of conversion *α_c_* (note that the “*A*·e^−*E*/R*T*^” term is also a denoted rate constant, *K*). For the macroscopically monitored crystal growth processes, the two most commonly used *f*(*α_c_*) functions are the semi-empirical flexible autocatalytic Šesták-Berggren [48] (AC) model (Equation (2)) and the nucleation-growth Johnson–Mehl–Avrami [43,44,45,46] (JMA) model (Equation (3)):

(2) $${f(\alpha_{c})}_{AC}=\alpha_{c}^{M}{\left(1-\alpha_{c}\right)}^{N}$$

(3) $${f(\alpha_{c})}_{JMA}=m\left(1-\alpha_{c}\right){\left[-ln\left(1-\alpha_{c}\right)\right]}^{1-\left(1/m\right)}$$

where *M*, *N*, and *m* are the kinetic exponents of the given models. One of the recently developed popular approaches to the enumeration of Equation (1) is the combination of the Kissinger plot-based determination of the (potentially temperature-dependent) activation energy, *E* [47], and the consequent application of the single-curve multivariate kinetic analysis (sc-MKA) [59]. The Kissinger equation can be expressed as follows:

(4) $$ln\left(\frac{q^{+}}{T_{p}^{2}}\right)=-\frac{E}{RT_{p}}+const.$$

where *T_p_* corresponds to the temperature of the maximum of the kinetic peak (technically, a similarly accurate evaluation can also be utilized for any other characteristic process-related temperature that is associated with a constant *α_c_* value). With the knowledge of *E*, a curve-fitting procedure based on Equations (5) and (6) can be applied to each individual DSC curve—the non-linear optimization (usually based on the Levenberg–Marquardt algorithm) is employed to determine the kinetic parameters from Equations (1)–(3), while *E* is fixed at the value determined from the Kissinger plot:

(5) $$RSS=\sum_{j=1}^{n}\sum_{k=First_{j}i}^{Last_{j}}w_{j,k}{\left(Y\exp_{j,k}-Ycal_{j,k}\right)}^{2}$$

(6) $$w_{j}=\frac{1}{{\left|{\left[d\alpha /dt\right]}_{\max}\right|}_{j}+{\left|{\left[d\alpha /dt\right]}_{\min}\right|}_{j}}$$

where *RSS* is the sum of squared residue, *n* is a number of measurements, *j* is an index of the given measurement, *First_j_* is the index of the first point of the given curve, *Last_j_* is the index of the last point of the given curve, *Yexp_j,k_* is the experimental value of the point *k* of curve *j*, *Ycal_j,k_* is the calculated value of the point *k* of curve *j*, and *w_j_* is a weighting factor for curve *j*.

#### 3.1.1. Griseofulvin

**Figure 7:**
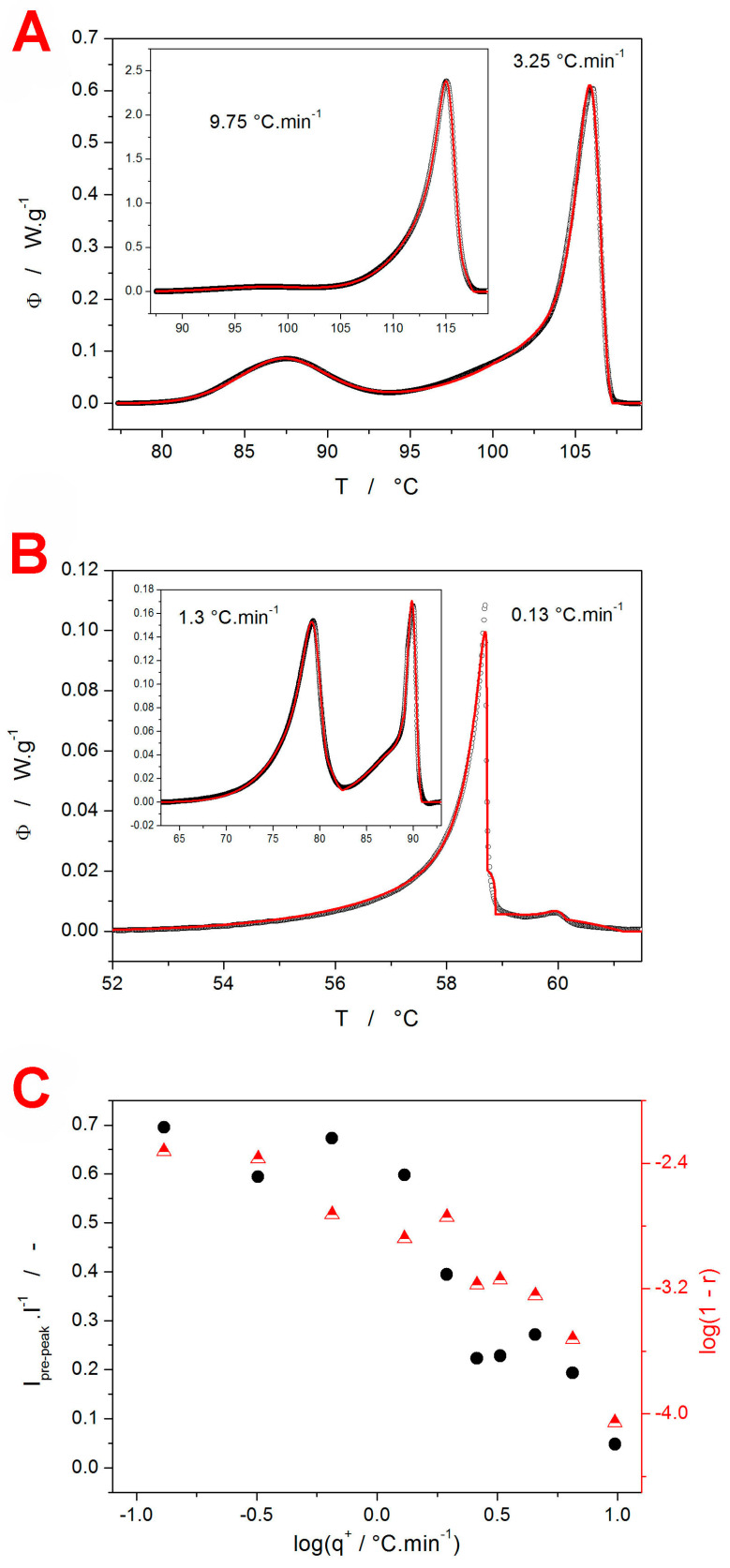
(**A**,**B**) Examples of the experimental GRIS DSC curves (points) fit with the sc-MKA method (using the reaction mechanism consisting of three to four independent AC processes; red line). (**C**) Relative crystallization enthalpy of the GRIS crystallization pre-peak (normalized with respect to the overall Δ*H*; filled circles and left Y axis) and the overall correlation coefficients (in the form of log(1-*r*); half-filled triangles and right Y axis), as obtained during the sc-MKA processing of the DSC crystallization data.

The above-described procedure was utilized to describe the GRIS DSC data obtained at different *q*^+^—several examples of these fits are shown in Figure 7A (higher *q*^+^) and Figure 7B (lower *q*^+^). The formal reaction/transformation mechanism consisted of three to four independent AC processes (mathematically expressed as a sum of three to four combinations of Equations (1) and (2). At a higher *q*^+^ (1.95–9.75 °C·min^−1^), the crystallization pre-peak could be formally described by a single AC process; at a lower *q*^+^ (0.13–1.3 °C·min^−1^), the pre-peak had to be modeled as a sum of a slower process (accounting for the prolonged onset of the peak) and a rapidly developing secondary process (accounting for the tip of the kinetic peak). A similar approach to summing the two AC kinetic processes had to be used for the main crystallization peak at all *q*^+^. This approach is generally common for the macroscopically monitored crystallization processes in amorphous drugs, where the growth of different polymorphs can proceed on the surface or in the volume, propagating via several different mechanisms, manifesting into different morphologies, and originating from the nuclei formed during the preparation of the amorphous phase, from mechanical defects, from nuclei formed during the heating itself, either in the amorphous matrix (still a primary nucleation) or on top of the already existing crystalline phases (secondary nucleation). The mathematical formalism used is, therefore, not necessarily a reflection of the true crystal growth mechanisms; it is, rather, a reaction/transformation model that best describes the kinetic features of the data, which can be consequently used for accurate predictions of the crystallization behavior. From this point of view, the main finding is the fact that the kinetic modeling was possible to perform using the independent processes (note that, for the crystallization from the amorphous phase, a similar mathematical model is also derived for the subsequent reactions; i.e., the secondary nucleation/crystal growth is not ruled out).

The mathematical details for the set of differential kinetic equations and heat balance conditions are given in the Supplementary Materials online, together with the full set of the final optimized kinetic parameters. In accordance with the concept of the sc-MKA method, the activation energies (to be fixed during the non-linear optimization) were obtained from the Kissinger dependencies shown in Figure 3A: for the crystallization pre-peak, *E*_1_ = 110.4 ± 2.8 kJ·mol^−1^, and for the main crystallization peak, *E*_2_ = 71.8 ± 3.1 kJ·mol^−1^. The two main conclusions associated with these results are visualized in Figure 7C. The kinetic description was, indeed, of high quality (with the worst correlation coefficient being *r* > 0.995 and the average correlation coefficient being *r_aver_* > 0.998). In the case of low-*q*^+^ data, the higher inconsistencies were caused by the possible imprecise subtraction of the thermokinetic background (the tangential area-proportional baseline [60] was used), which can overestimate the area of the peak tails for the main crystallization process under these conditions. This overestimation is then at least partially reflected in the proportional area of the crystallization pre-peak Δ*H_1_*/Δ*H* quantity (depicted in Figure 7C), where the true values for *q*^+^ = 0.13 and 0.32 °C·min^−1^ should probably be closer to 0.9 and 0.8, respectively. For example, the DSC curve for 0.13 °C·min^−1^ (Figure 7B) shows the “main” crystallization peak, the proportional area of which is ~1% of the overall crystallization enthalpy, and only the underlying background represents the additional ~29% of Δ*H* (the exact quantifications are included in the Supplementary Materials online). Nonetheless, the basic trend of switching between the two crystal growth processes (the GC crystallization and the consequent surface or bulk secondary growth) with decreasing a *q*^+^ (resulting in a longer time spent below *T_g_*) is clearly apparent.

Concerning the IRM technique being considered an alternative to the DSC measurements, in the cases akin to the present GRIS data, it was shown that IRM is capable of detecting GC growth with similar effectiveness as the DSC instrumentation (see Figure 3B). The added value of the IRM technique is the potential direct identification of the polymorphic phase, which could be overrun or re-crystallized during the ex situ DSC experiment (RS measurement of the partially DSC-crystallized sample). However, with IRM being a primarily surface-based technique and the corresponding confocal depth analyses being time-demanding, the DSC clearly surpasses IRM in the extent of the information about the kinetics and nature of the concurrent processes (apart from the initiation of the first amorphous-to-crystalline transformation).

#### 3.1.2. Indomethacin

Whereas the data for GRIS (Section 3.1.1) were a demonstration of an approach to modern kinetic analysis that is rather standard nowadays (the Kissinger method paired with sc-MKA optimization), and the main novelty was the detection and kinetic analysis of the GC crystal growth, in the case of IMC data, the very recently developed approach [42] to the evaluation of the *T*-dependent crystallization kinetics will be utilized and discussed with regard to the potential employment of the IRM technique. In particular, the implementation of the *T*-dependent activation energy into the sc-MKA method will be introduced. As was shown in Figure 6B, the Kissinger dependence obtained for IMC is clearly curved, indicating the kinetics inconsistent with the full-range application of the Kissinger equation. However, if we consider differentially small segments of the dependence, these can be approximated using the linear function, the slope of which is consistent with the derivation of Equation (4). In practice, the curved Kissinger dependence can be fit with a suitable mathematical function (a second-order polynomial in the present case), the derivation of which can be used to calculate the *E*–*T* dependence according to Equation (4).

**Figure 8:**
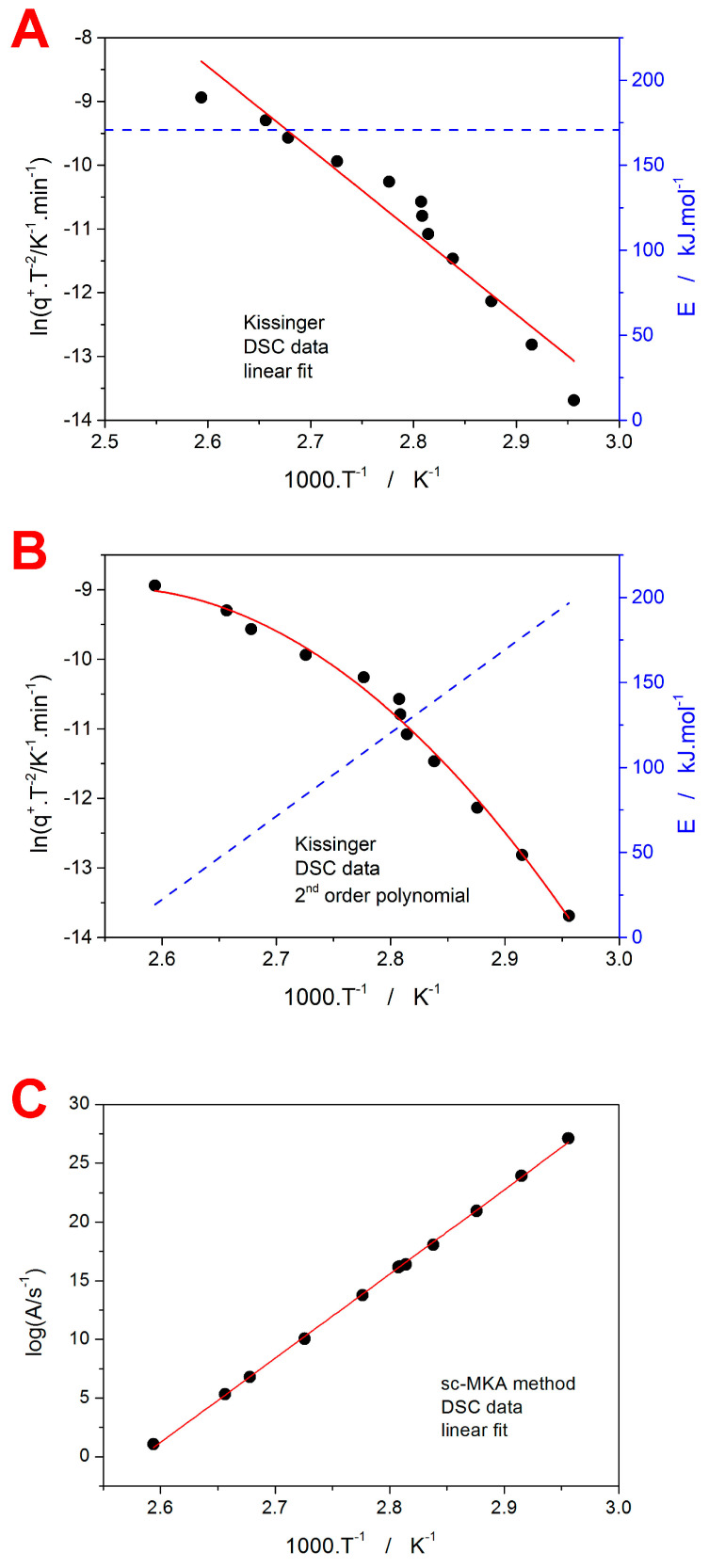
(**A**) Kissinger plot constructed for the IMC *T_p_* data (points) fit with the linear dependence (the solid line). The dashed line paired with the right-hand Y axis shows the corresponding temperature dependence of the activation energy, *E*. (**B**) Kissinger plot constructed for the IMC *T_p_* data (points) fit with the second-order polynomial dependence (the solid line). The dashed line paired with the right-hand Y axis shows the corresponding temperature dependence of the activation energy *E*. (**C**) Values of the pre-exponential factor *A* determined by the sc-MKA method for the *E*–*T* dependence shown in (**B**). The A values were determined at temperatures corresponding to the experimental points from (**B**). The solid line shows the almost perfect fit with a linear dependence, demonstrating the *E*–*A* compensation effect.

**Figure 9:**
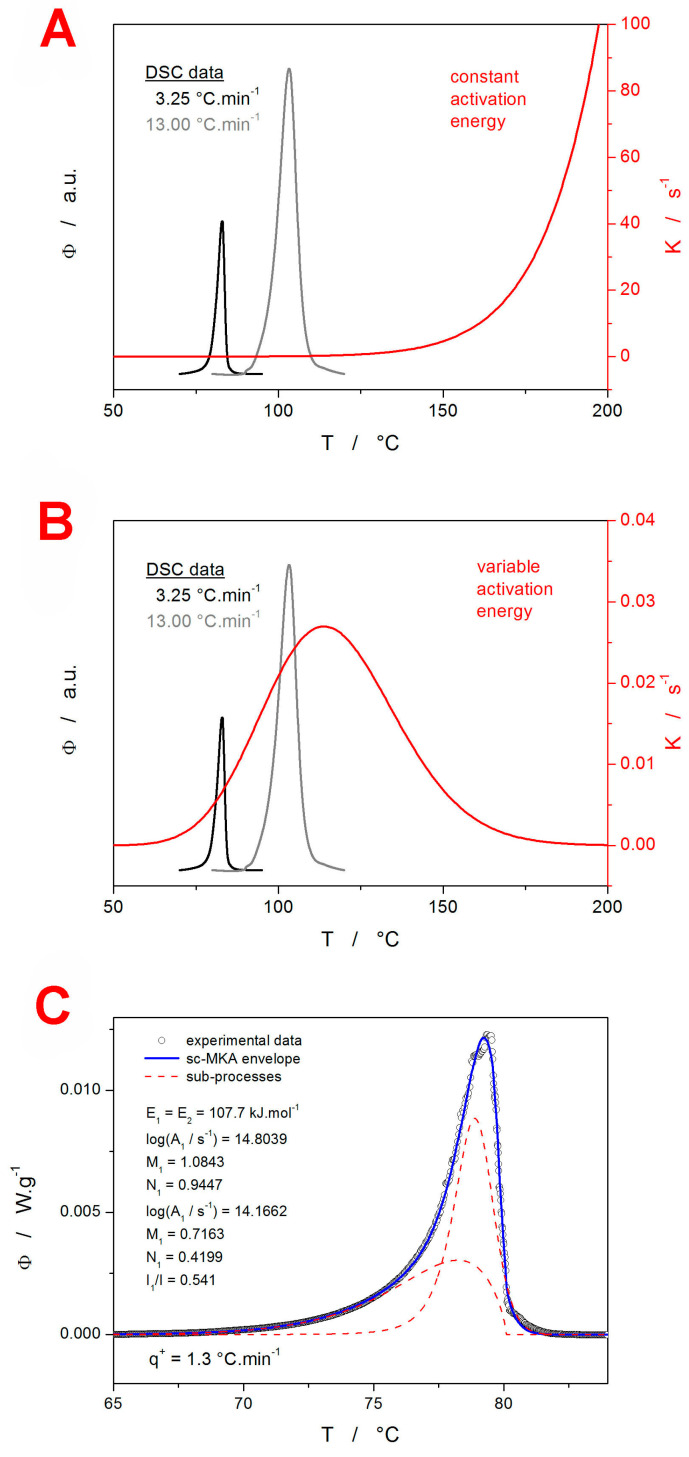
(**A**) Temperature dependence of the rate constant (right-hand axis) calculated using the constant *E* and *A* values determined for the crystallization of IMC (see Figure 8A); the graph depicts two selected DSC crystallization peaks for comparison. (**B**) Temperature dependence of the rate constant (right-hand axis) calculated using the variable *E* and *A* values determined for the crystallization of IMC (see Figure 8B,C); the graph depicts two selected DSC crystallization peaks for comparison. (**C**) Example of the IMC crystallization peak processed by means of the sc-MKA method.

In Figure 8A,B, the difference between the linear and polynomial fits of the IMC Kissinger plot is shown, including the corresponding *E*–*T* dependencies. It is clear that, for the present IMC data, the linear approximation of the Kissinger dependence would be an unallowable oversimplification. By utilizing the *E* values determined based on the *E*–*T* dependence from Figure 8B for the particular *T_p_*s, the sc-MKA method can be modified for the varying *E*. In such a case, the pre-exponential factor *A* needs to be adjusted as well; the corresponding log*A*–*T* dependence is shown in Figure 8C. By implementing the *E*–*T* and *A*–*T* dependencies into Equation (1), the rate constant *K* = *A*·e^−*E*/R*T*^ can be calculated [42]. Whereas, for the constant value of *E* (Figure 8A), the rate constant exponentially increases with *T* (see Figure 9A), for the present combination of *E*–*T* and *A*–*T*, the *K*–*T* dependence exhibits a maximum in between *T_g_* and *T_m_* (see Figure 9B). As was shown in [42], if the crystallization proceeds near or above the onset inflection point of the *K*–*T* dependence (as happens for the present data measured at 13 °C·min^−1^), the asymmetry of the kinetic peak changes to more positive values (i.e., the peak skews to lower *T* and α values). This is indeed true for the present data, which not only supports the claim of the *K*–*T* dependence determination accuracy but also verifies the practicality of the conclusions introduced in the (theoretical) study [42]. Note that even larger skewing to the low *T* and α values occurred at *q*^+^ = 19.5 °C·min^−1^, where the DSC crystallization peak exhibited fully positive asymmetry—the raw DSC curve is shown in the Supplementary Materials online. To complement the GRIS study, the IMC data were also processed in terms of the sc-MKA method—with both constant and variable *E*&*A*. In practice, the description was similar for both options because, in sc-MKA, E is compensated by *A* (as is implied from Figure 8B,C). Similarly to the GRIS DSC data (Figure 7A,B), the crystallization of IMC also needs to be mathematically described by two independent (or concurrent) AC processes—an example of their distribution within the overall heat flow envelope is shown in Figure 9C (qualitatively similar curve-fitting results were obtained for all other GRIS and IMC DSC crystallization peaks, as was already discussed in Section 3.1.1).

Regarding the potential utilization of the IRM technique for the related type of kinetic calculations described above, the key feature, i.e., the curvature of the ln(*q*^+^·*T*^−2^)-*T*^−1^ dependence obtained for the DSC crystallization peaks (see Figure 6B), was also relatively accurately reflected through the IRM data. Hence, qualitatively similar estimates of the *K*–*T* dependence (as shown in Figure 9B for the DSC data) can also be derived based on the IRM measurements. Nonetheless, similar to the case of GRIS, the present precision of the IRM-measured α-T dependencies (Figure 2C for GRIS and Figure 5C for IMC) is not sufficient for the consistent and reproducible kinetic analyses, such as those demonstrated, e.g., in Figure 7 and Figure 9C. A further extended evaluation of the IRM technique’s performance will be given in the concluding section.

### 3.2. Theoretical Assessment of the In Situ Collection of Raman Spectra

In general, the in situ collection of the Raman spectra during linear heating can be realized in two ways. In the first way, a step-like temperature program is employed, where the collection happens during isothermal annealing at different temperatures, in between which relatively fast heating is performed that should be negligible in duration compared to the isothermal holds. The second approach to the spectra collection is realized simply during linear heating, when the spectra are continuously collected, one after another, in time segments dictated by the measurement conditions. The consequences of the former (step-like) temperature program on the determination of the amorphous phase crystallization kinetics were analyzed in detail in [32] (the analyses were performed with X-ray diffraction patterns being considered/collected, but exactly similar conclusions are also valid for the collection of the Raman spectra): (1) the asymmetry or shape of the crystallization signal/peak does not significantly influence the errors associated with the kinetics evaluation; (2) the largest errors are obtained for longer isothermal annealing, larger temperature steps between the annealing phases, and processes with high activation energy, *E*; (3) and the model-free kinetic parameters (*E* and *A*) exhibit larger errors compared to the model-based characteristics, and the non-linear optimization is the most accurate method for the determination of the in situ measured step-like crystallization kinetics. The potential distortions associated with the second way of collecting the data during in situ linear heating Raman measurements were, however, not yet addressed in the literature (to the best knowledge of the authors).

**Figure 10:**
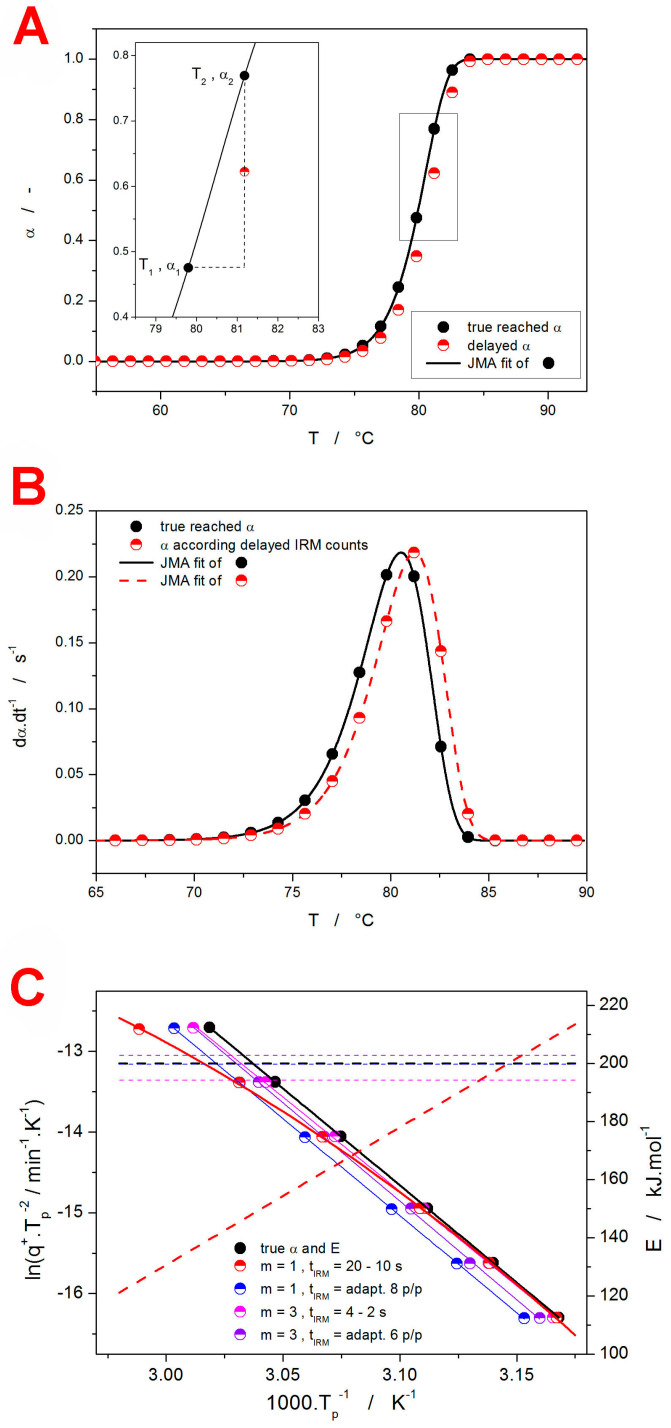
(**A**) Filled-in points: α-T dependence simulated for *E* = 200,000 kJ·mol^−1^, ln(*A*/s^−1^) = 62.3, *m* = 3, *q*^+^ = 1 °C·min^−1^, and *t_IRM_* = 83 s. Half-filled-in points: the α-*T* dependence delayed by subtracting *t_IRM_*/2 (41.5 s). The inset shows the construction of the delayed data point from the original α-*T* dependence according to Equation (8). (**B**) The derivative dα·d*t*^−1^-*T* dependencies corresponding to the similar types of points from Figure 10A. The lines indicate the JMA fits of these dependencies. (**C**) Points: Kissinger dependencies constructed for the series of theoretically simulated kinetic peaks with *E* = 200,000 kJ·mol^−1^, ln(*A*/s^−1^) = 62.3, and *q*^+^ = 0.5, 1, 2, 5, 10, and 20 °C·min^−1^; the *m* and *t_IRM_* values are listed in the legend. Solid lines: linear or polynomial fits of the Kissinger dependencies. Dashed and dotted lines (matched with the right-hand Y-axis): *E*–*T* dependencies calculated based on the fits of the Kissinger dependencies.

In the present section, the findings presented in [32] will be adapted to theoretical simulations exploring the accuracy of the kinetic calculations applied to the linear heating temperature program within the in situ Raman microscopy measurements. In such a case, the fundamental idea is that the Raman counts collected for each spectrum represent an average of a progressively increasing α. This is demonstrated in the inset of Figure 10, where the filled-in black points indicate the temperatures reached at the end of each cycle of the Raman spectra collection; note that the Raman spectra were collected as a sum of several scans:

(7) $$t_{IRM}=t_{s}\cdot n_{s}$$

where *t_s_* and *n_s_* are the time of one scan and the number of scans summed into one spectrum, respectively; only the overall time of the spectrum collection, *t_IRM_*, will be considered in the calculations shown in the present section—the corresponding reasoning and discussion will be introduced at the end of this section. As is shown in the inset of Figure 10A, as the sample temperature changes from *T*_1_ to *T*_2_ during the spectrum collection (lasting *t_IRM_*), the degree of conversion α (corresponding to the sample crystallinity in the present case) changes from α_1_ to α_2_. This means that, in the first seconds of such collection, the collection rate of the Raman counts corresponding to the crystalline phase is close to that characteristic for α_1_, and at the end of *t_IRM_*, a higher intensity of the crystalline phase Raman counts (characteristic for α_2_) is obtained. The best approximation of this α averaging over *t_IRM_* is is then expressed as a so-called delayed degree of conversion, α_d_, corresponding to the true α reached at the following time:

(8) $$t_{IRM}/2=\left(t_{2}-t_{1}\right)/2$$

where indices 1 and 2 correspond to the same indices from the Figure 10A inset, where α_d_ is indicated by the red half-filled-in points. Note that α_d_ is attributed to *T*_2_ (corresponding to the end of the spectrum collection). An example of the temperature shift between the true degree of crystallinity, α, reached within the sample and the delayed α_d_ (measured using the spectrometer) is shown in Figure 10B for the amorphous-to-crystalline process simulated in terms of the JMA kinetics. The derivation of these α-*T* signals leads to the characteristic dα·d*t*^−1^-*T* kinetic peaks (akin to the information obtained, e.g., from DSC) that can be processed by means of the standard methods of kinetic analysis (Equations (1)–(6)).

In the present section, different types of non-isothermal crystallization kinetic data (akin to those depicted in Figure 10B) were first simulated in terms of the JMA model (defined by *E*, *A*, *m*, and *q*^+^) and then processed/recalculated to imitate the IRM delay of the dα·d*t*^−1^-*T* signals (the variable was *t_IRM_*; the corresponding delayed α_d_ was always taken for *t_IRM_*/2). The delayed dα_d_·d*t*^−1^-*T* data were consequently evaluated using the sc-MKA method, with the delayed activation energy value, *E_d_*, being similar to the one input into the original undistorted simulation (*E_d_* = *E*). With a fixed *E_d_*, the unconstrained freely optimized variables were the ln*A_d_* and *m_d_*. In the first round of simulations, the influence of the IRM delay on the deviation of the peak asymmetry (loosely expressed by the JMA kinetic exponent *m*) was tested for two sets of fixed parameters: *E* = 50 kJ·mol^−1^ & ln(*A*/s^−1^) = 10, *E* = 200 kJ·mol^−1^ & ln(*A*/s^−1^) = 70. Within the tested range of *m* = 1–3.5, the delay-caused error in the determination of the JMA kinetic exponent was |*m* − *m_d_*| ≤ 0.05. By changing the *t_IRM_*, the tests were also performed with respect to the varying density of points; scenarios with the 3–30 p/p (points per peak, where the peak onset and endset were defined at 5% of the peak height) were explored. Similarly negligible deviations in the determination of m were also obtained for all other performed tests (described below), which indicates that the JMA kinetic exponent evaluation (and, by extension, the asymmetry of the kinetic peaks) is, in practice, not influenced by the delayed recording of the crystallization signal during the IRM measurements.

The second round of tests was aimed at exploring the delay-induced errors within the *E*–*A* compensation framework. For the constant JMA kinetic exponent *m*, the position of the peak maximum (*T_p_*), and the density of points (7 p/p), a series of *E* and *A* pairs was derived (*E* values were arbitrarily chosen, and *A* values were determined to fulfill the *T_p_* = const. condition)—the data are shown in Table 3. Despite the *m_d_* value changing again only very slightly, an interesting feature of *A_d_*/*A* = const. was observed. Since the first round of tests exhibited a similar feature for varying the kinetic exponent *m*, the *A_d_*/*A* magnitude can clearly depend only on *q*^+^ or the density of points. Note that *A_d_*/*A* equals “ln*A_d_* − ln*A*”; i.e., it expresses the effective shift of the kinetic peaks along the temperature/time axis.

To explore this feature in detail, a third round of tests was performed, consisting of four series of simulations performed at different *q*^+^ values and either a constant *t_IRM_* or a density of points (p/p)—the parameters input into the simulations, as well as the results of the sc-MKA fits of the dα_d_·d*t*^−1^-*T* data, are listed in Table 4. Whereas the *m_d_* values are again practically identical to the *m* values input into the simulations, the *A_d_*/*A* ratio indeed changed in certain scenarios. Evidently, the key aspect is the density of points and its potential variability. In the cases where *t_IRM_* was adjusted (increased with a decreasing *q*^+^) so that the density of points (p/p) remained constant, the temperature shift of the IRM-measured kinetic data also remained the same for all *q*^+^ (*A_d_*/*A* = const.). On the other hand, with a constant *t_IRM_*, the density of points increases with a decreasing *q*^+^, making the temperature shift smaller at a low *q*^+^ and ultimately resulting in *A_d_*/*A* being limited to 1 at an infinitely slow *q*^+^. This is demonstrated even better via the Kissinger plots (constructed according to Equation (4)) that visualize the data from Table 4—see Figure 10C. It is clear that, with the constant number of points per peak (adapted *t_IRM_*), the whole Kissinger dependence is only shifted to higher temperatures without any major change in its slope (leading to *E_d_* ≈ *E*). On the other hand, by setting *t_IRM_* = const., the shift of *T_p_* (and, naturally, the whole kinetic peak) to higher temperatures increases with *q*^+^, which results in a change in the slope of the Kissinger dependence and, thus, *E_d_* ≠ *E*. In the case of *t_IRM_* = 4 s, the delay of α_d_ vs. α is rather small even at a high *q*^+^; thus, the distortion is negligible, and the Kissinger dependence can be fit linearly with a minimum error. On the other hand, for *t_IRM_* = 20 s, the delay of the kinetic signal is already significant, and it progressively increases with *q*^+^ (↑*q*^+^ is associated with a low effective density of points, which in turn causes a larger decrease in α_d_). In such a case, the linear fit would be a great oversimplification (leading to *E_d_* ≈ 167 ± 6 kJ·mol^−1^). Instead, the temperature dependence of activation energy *E_d_*(*T*) needs to be determined through, e.g., the derivation of a polynomial fit of the Kissinger dependence—as shown in Figure 10C. Obviously, the smallest deviation of *E_d_* from *E* is obtained at a low *q*^+^, where a high density of points can be achieved regardless of the rapidity of the crystallization process.

To generalize the above-described theoretical simulations and their kinetic analysis, several aspects need to be considered. First, regarding the question of the asymmetry of the kinetic peak, the above-described calculations were performed for the JMA kinetics, which is characteristic of a strictly defined negative asymmetry (skewing to a higher α). As was already suggested earlier [32], asymmetry has a rather negligible impact on the distortions caused by the prolonged in situ collection of data points. This was indeed also verified for the present simulated IRM measurements by performing tests for a significantly positively asymmetric Šesták–Berggren kinetic peak: the more rapid onset of the kinetic peak even led to a small decrease in the errors associated with the delayed recording of the true α—especially in the case of *E* (i.e., *E_d_*) determination. The second generalizing aspect regards the *T_2_* ~ α_d_ ≈ α (*t_IRM_*/2) correspondence—see Figure 10A for details. From the purely kinetic point of view, the α_d_ ≈ α (*t_IRM_*/2) equality is valid only if the kinetic route between *T_1_* and *T_2_* can be described linearly (which is true only for a large density of points). For the convex/concave courses of the α-*T* dependence (occurring as a significant feature at lower densities of collected data points), the α_d_ is overestimated/underestimated, respectively, using *t_IRM_*/2. Since the absolute majority of the crystallization processes exhibit a sigmoidal α-*T* dependence with both a first convex part and then a concave part, the constant value of the divisor (universally approximated as 2) leads to a slight distortion in the peak asymmetry, which slightly skews the asymmetry of the kinetic peak to higher α values—this is indeed noticeable from the universally higher m values (*m_d_* > *m*; see Table 3 and Table 4). The real impact is, however, negligible. The second part of this question is the exact value of the divisor in the *t_IRM_*/2 term. For the divisor values closer to unity (between 1 and 2), the delays would be smaller, and the potential errors of *A_d_* and *E_d_* (in comparison with their true counterparts) would be smaller, and vice versa. Nonetheless, for a combination of a standard kinetic process and a Raman microscope, the divisor value of 2 should be very close to the actual physico-chemical manifestation of the procedure. The third important aspect of the universality of the presently introduced calculations is the boundaries of the parameters input into the simulations. From the material point of view, the JMA kinetic exponent between 1 and 3.5 encompasses the absolute majority of the crystallization processes [61]. Also, the activation energies between 50 and 200 kJ·mol^−1^ cover a large majority of the crystallization processes in amorphous drugs—see, e.g., the recent particle size-oriented crystallization studies on amorphous indomethacin [62] and griseofulvin [49]. The main experimental variables are *q*^+^ (the range of 0.5–20 °C·min^−1^ indeed covers the absolute majority of the related studies) and *t_IRM_*. In the case of the latter, a large portion of the measurements can be performed with *t_IRM_* ensuring at least 5–20 p/p density, which is the range mainly focused on in the present section. Note that larger densities of collected points are, of course, achievable at a lower q^+^, but without proper automation, their processing is tedious and unnecessary. On the other hand, for the materials with a weak Raman signal (resulting in a mandatory high *t_IRM_*), only a low *q*^+^ should be considered for the IRM measurements to preserve the sufficient density of the collected points (at least 5–10 p/p; the lower the measurement precision, the higher the points’ density).

## 4. Materials and Methods

The amorphous APIs were prepared from their as-purchased crystalline forms: the γ polymorph of IMC (purity > 99 %; Sigma-Aldrich, Prague, Czech Republic) and the I. polymorph of GRIS (purity > 97 %; Sigma-Aldrich, Prague, Czech Republic). In particular, approx. 1 g of the crystalline powders was poured into glass vials, which were then placed in oil baths at temperatures slightly above the drugs’ melting points (180 °C for IMC and 215 °C for GRIS); after the liquefaction of the APIs, the vials were quenched in cold water. For both APIs, the cooling rate was more than sufficient for a yellowish, defect-less ingot of glassy material to be formed. The amorphous APIs were lightly ground using an agate mortar and pestle to produce the 20–50 μm powders—the particle size fraction was separated using sieves with a defined mesh (Retsch, Haan, Germany). Note that this specific particle size fraction was selected due to the relative ease of preparation (not needing overly rough grinding and potentially causing the partial formation of a crystalline phase) together with the certainty of a large amount of the mechanical defects being present on each grain of the powdered materials. Low-molecular glasses such as IMC and GRIS crystallize dominantly from surface-mechanical defects (e.g., micro-cracks, edges, or tips) [33,34,35,36,37]. The large amount of defects on the surface of the fine powder grains ensures a more or less homogeneous distribution of the crystallization centers on the whole surface of each grain; this increases the reproducibility of the IRM measurements, which are always focused only on a very small spot of an arbitrarily selected grain. The prepared powders were stored in dark, dry air at −5 °C. The absolute majority of the measurements were performed within a week of the samples’ preparation.

The in situ Raman microscopy (IRM) measurements were realized using a DXR2 Raman microscope (Thermo Fisher Scientific, microscope body from Olympus, Prague, Czech Republic) equipped with a 785-nm excitation diode laser (laser spot size: 3.1 μm at ×10 magnification) and a CCD camera. The experimental conditions for the Raman measurements were 20 mW laser power on the sample, a 1–10 s duration for a single scan, and 5–100 scans summed in one spectrum (the values were adjusted to obtain at least 10 full high-quality Raman spectra over the expected temperature range of the amorphous-to-crystalline transformation). The Raman microscope was calibrated daily for the wavelength, Raman shift, and optical path alignment. The temperature program was realized using a heated stage CCR1000 (Linkam, Redhill, UK); the measurements were performed as linear heating steps at 25–150 °C with the heating rates, *q*^+^, equal to 0.13, 0.32, 0.65, 1.30, 1.95, 2.60, 3.25, 4.55, 6.50, and 9.75 °C·min^−1^ (the in-programmed integer/rounded q^+^ values were recalculated to a true q^+^ using temperature calibration). The temperature profile of the stage was calibrated on the melting of pure metals (Ga, In, and Zn).

As a supplemental and reference experimental technique to IRM, differential scanning calorimetry (DSC) was used. In particular, the heat flow Q2000 instrument (TA Instruments, New Castle, USA) equipped with an autosampler, an RCS90 cooling accessory, and T-zero technology was utilized. The DSC was calibrated with the H_2_O, In, and Zn standards. The measurements were performed in hermetically sealed, low-mass DSC pans (i.e., static air atmosphere was applied); the sample masses varied between 1 and 3 mg (accurately weighed to 0.01 mg) based on the associated *q*^+^: the higher the *q*^+^, the lower the mass to keep the intrinsic thermal gradients roughly equal. Similar to IRM, the DSC measurements were also performed as linear heating steps in 25–250 °C (an increased upper temperature limit to record also the melting process) with the same heating rates, *q*^+^ = 0.13, 0.32, 0.65, 1.30, 1.95, 2.60, 3.25, 4.55, 6.50, and 9.75 °C·min^−1^. Reproducibility was checked for 20% of randomly selected measurements.

## 5. Conclusions

The performance of in situ Raman microscopy was compared with that of differential scanning calorimetry (a standard technique in the field) with regard to the monitoring of crystal growth kinetics in low-molecular glasses: amorphous GRIS and IMC. For GRIS, a remarkably intense rapid GC growth was found to dominate at *q*^+^ < 1 °C·min^−1^; this process was successfully monitored (with similar accuracy for its detection) using both techniques—DSC and IRM. The IRM approach (evaluating the temperature corresponding to the highest conversion rate between the amorphous and crystalline phases) reproduced the kinetic features associated with the onset of the DSC-recorded crystallization peak well. However, as a surface technique, single-spot IRM can cover only the initial stage of the crystal growth process. In the case of IMC, the non-monotonous temperature dependence of the rate constant, as well as the potential identification of different polymorphic phases, was at play. Whereas the IRM-estimated *E*–*T* dependence was quite similar to the one accurately determined from the DSC-measured Kissinger data, the recognition of the different polymorphic phases was proven at its fundamental basis, but the reproducibility of the IRM polymorphic kinetics was found to be very low. Additionally, in neither case (GRIS or IMC data) was the precision or reproducibility of the IRM measurements and evaluations, i.e., a significant influence of the choice of the borderline spectra representing the amorphous and crystalline states in each given case, sufficient to imitate the extent of information collected via DSC.

The main recommendations regarding the IRM instrumentation and experimental conditions can be summarized as follows. The first key aspect for accurate IRM measurements is the securing of a sufficiently precise temperature cell—the high-temperature catalytic CCR1000 cell was found not to be suitable; a significantly better option would have been, e.g., the low-temperature THMS600 stage (also Linkam, Redhill, UK). The second crucial aspect is temperature calibration—an independently calibrated thermocouple appears to be a significantly more precise option compared to direct calibration based on the visual identification of the melting of pure metals. Also, at least a three-point *T* calibration should always be performed for these temperature stages since a significantly non-linear *T_true_*–*T_measured_* dependence appears to be rather common for these microscope heating stages. The third important aspect is the proper identification of the initial and final stages of the amorphous-to-crystalline transformation. In this regard, the best approach was to associate each particular IRM measurement with its own pair of the “fully amorphous” and “fully crystalline” spectra to suppress the variation between individual sets of experimental conditions. The fourth aspect considered was the Raman spectra collection method—theoretical simulations have shown that it is better to adjust the *t_IRM_* to the *q*^+^ so that the density of the collected points per kinetic effect is roughly similar. The fifth and most crucial aspect was the too-small size of the spot (a few μm^2^) from which each spectrum was collected. This led to acceptable reproducibility only in the case of the dominant polymorphic phase, partially negating the main advantage of the RS technique (i.e., the easy recognition of individual polymorphs). In this regard, a groundbreaking improvement would be the employment of the modern fast Raman mapping techniques, which (using multichannel detectors and advanced stage-movement instrumentation) can collect Raman spectra with high spatial resolutions from large sample areas. Effectively collecting Raman spectra from a large portion of a sample surface would not only allow the polymorphic distribution of the surface crystalline phase to be mapped but also gain added information about the crystallization mechanisms of each polymorph (the location and type of the crystallization centers investigated via depth profiling), and even the reliable recognition of potential recrystallization events could be attempted. In addition, fast Raman mapping would also lead to a major improvement in reproducibility and accuracy for the measurements, as the irregularities in the crystal growth rate detection (manifesting through the achieved degree of crystallinity) caused by temperature fluctuations or the non-homogoneous distribution of the crystallization centers on the sample surface would be smoothed out.

To conclude, we note that the standard IRM can be considered a suitable supplementary technique to the DSC, as it provides information about the local presence of polymorphic forms and can detect certain surface crystallization processes (e.g., crystal growth from pre-existing defects, such as micro-cracks and edges) early if properly focused on the appropriate spot. The microscopic nature of IRM is, however, also its largest disadvantage, as it cannot provide highly reproducible information (a signal averaged over a large surface area) about the detected changes. In this regard, only the employment of the fast Raman mapping functions could advance the performance of IRM above that of calorimetric measurements.

## Figures and Tables

**Table 1:** Characteristic temperatures and enthalpies evaluated from the DSC curves measured for the amorphous 20–50 µm GRIS powder (Figure 1): glass transition temperature, *T_g_*; temperature corresponding to the initiation of the crystallization process, *T_ini_*; temperature corresponding to the maximum of the crystallization pre-peak, *T_p_*_1_; overall crystallization enthalpy, Δ*H_c_*; temperature corresponding to the maximum of the main crystallization peak, *T_p_*_2_; onset temperature for the melting peak, *T_m,ons_*; peak temperature for the melting peak, *T_m,p_*; enthalpy of melting, Δ*H_m_*. The T_g_ values presented in boldface were calculated using the *T_g_* value determined from the *q*^+^ = 9.75 °C·min^−1^ curve, and the activation energy for the structural relaxation process Δh^*^ = 379 kJ·mol^−1^.

*q* ^+^	*T_g_*	*T_ini_*	*T_p_*_1_	Δ*H_c_*	*T_p_*_2_	*T_m,ons_*	*T_m,p_*	Δ*H_m_*
°C·min^−1^	°C	°C	°C	J·g^−1^	°C	°C	°C	J·g^−1^
0.13	**68.3**	52.6	58.7	48.2	59.9	203.7	209.0	84.3
0.32	**70.6**	55.3	67.0	55.7	68.7	206.0	211.4	88.4
0.65	**72.5**	62.4	74.5	54.8	79.8	208.2	213.7	107.4
1.3	**74.3**	65.7	79.1	55.8	90.0	211.0	214.8	104.4
1.95	**75.4**	70.9	82.9	53.2	97.7	212.7	215.6	111.0
2.6	**76.2**	77.4	84.8	50.8	101.1	213.0	215.9	113.8
3.25	**76.8**	81.3	87.5	48.8	106.0	214.1	216.6	115.9
4.55	**77.7**	82.8	88.9	60.8	107.1	214.3	216.8	124.1
6.5	**78.6**	85.9	93.7	57.2	113.1	215.0	217.4	116.7
9.75	79.7	89.4	97.6	63.5	115.0	215.7	217.9	123.0

**Table 2:** Characteristic temperatures and enthalpies evaluated from the DSC curves measured for the amorphous 20–50 µm IMC powder (Figure 4): glass transition temperature, *T_g_*; temperature corresponding to the initiation of the crystallization process, *T_ini_*; temperature corresponding to the maximum of the crystallization peak, *T_p_*; overall crystallization enthalpy, Δ*H_c_*; peak temperature for the first melting peak, *T_m,p_*_1_; enthalpy for the first melting peak, Δ*H_m_*_1_; peak temperature for the second melting peak, *T_m,p_*_2_; enthalpy for the second melting peak, Δ*H_m_*_2_.

*q* ^+^	*T_g_*	*T_ini_*	*T_p_*	Δ*H_c_*	*T_m,p1_*	Δ*H_m_*_1_	*T_m,p_*_2_	Δ*H_m_*_2_
°C·min^−1^	°C	°C	°C	J·g^−1^	°C	J·g^−1^	°C	J·g^−1^
0.13	28.4	63.6	65.1	55.6	-	-	155.3	103.5
0.32	28.7	68.0	69.9	61.5	-	-	156.2	104.8
0.65	31.0	72.6	74.6	56.8	148.4	16.3	157.2	95.6
1.3	32.1	76.9	79.2	61.4	149.3	32.0	157.1	87.5
1.95	33.6	79.7	82.2	59.0	148.9	39.3	156.8	72.8
2.6	34.9	79.9	82.9	62.0	150.6	35.3	157.3	80.9
3.25	35.4	79.9	83.0	60.1	152.0	65.4	158.5	57.6
4.55	36.1	84.2	87.0	56.8	152.3	76.0	158.7	41.8
6.5	37.9	89.9	93.7	58.7	152.9	73.1	159.0	29.5
9.75	38.9	95.2	100.3	65.2	153.1	68.4	159.2	27.4

**Table 3:** Second round of tests aimed at quantifying the distortions caused by the delayed collection of the Raman signal during the in situ measurements. The simulations were performed for *m* = 3, *q*^+^ = 1 °C·min^−1^, and *E*, *A*, and *t_IRM_* listed in the table. The quantities with a subscript “d” are the results of the sc-MKA fits (with a fixed true value of *E*) of the delayed IRM dα·d*t*^−1^-*T* dependencies.

E	ln(A/s^−1^)	t_IRM_	m_d_	ln(A_d_/s^−1^)	lnA − lnA_d_	A_d_/A
kJ·mol^−1^	°C	s	-	^-^	-	-
50	10	300	3.05	9.864	0.136	0.873
75	19	212	3.03	18.861	0.139	0.870
100	27.7	159	3.02	27.565	0.135	0.874
125	36.4	130	3.02	36.264	0.136	0.873
150	45	110	3.02	44.863	0.137	0.872
175	53.7	93	3.01	53.565	0.135	0.874
200	62.3	83	3.01	62.163	0.137	0.872

**Table 4:** Third round of tests aimed at quantifying the distortions caused by the delayed collection of the Raman signal during the in situ measurements. The simulations were performed for *E* = 200,000 kJ·mol^−1^, ln(*A*/s^−1^) = 70, and *q*^+^, *m*, and *t_IRM_* listed in the table. The quantities with a subscript “d” are the results of the sc-MKA fits (with a fixed true value of *E*) of the delayed IRM dα·d*t*^−1^-*T* dependencies.

*q* ^+^	*m*	*t_IRM_*	*m_d_*	ln(*A_d_*/s^−1^)	Density of Points
°C·min^−1^	-	s	-	^-^	p/p
20	1	20	1.020	69.257	4
10	1	20	1.010	69.620	7
5	1	20	1.005	69.806	15
2	1	20	1.002	69.920	36
1	1	20	1.001	69.959	71
0.5	1	20	1.001	69.979	135
20	1	10	1.010	69.627	8
10	1	19.7	1.010	69.627	8
5	1	39	1.010	69.623	8
2	1	95.5	1.010	69.622	8
1	1	188	1.010	69.621	8
0.5	1	354	1.009	69.6379	8
20	3	4	3.012	69.850	6
10	3	4	3.006	69.924	12
5	3	4	3.003	69.961	25
2	3	4	3.001	69.984	60
1	3	4	3.001	69.992	115
0.5	3	4	3.001	69.996	222
20	3	4	3.012	69.850	6
10	3	7.8	3.012	69.851	6
5	3	15.3	3.012	69.851	6
2	3	37.5	3.012	69.851	6
1	3	68	3.045	69.832	6
0.5	3	139	3.027	69.852	6

## Data Availability

The original data presented in the study are openly available at https://doi.org/10.6084/m9.figshare.27175326.v1 (accessed on 6 October 2024).

## Supplementary Materials

The following supporting information can be downloaded at https://www.mdpi.com/article/10.3390/molecules29194769/s1: Figures S1 and S2, depicting example full raw sets of Raman spectra obtained via IRM; Table S1, as part of a report on the sc-MKA results obtained for amorphous GRIS; Figure S3, depicting the DSC curve of amorphous IMC measured at 19.5 °C·min^−1^.
