# Supplementary material for: In Situ Raman Spectroscopy as a Valuable Tool for Monitoring Crystallization Kinetics in Molecular Glasses
Source: Molecules. 2024 Oct 9;29(19):4769. doi: 10.3390/molecules29194769 (PMC11478080; doi:10.3390/molecules29194769)
Supplement: Supplementary file 1 [file molecules-29-04769-s001.zip › molecules-3247339-supplementary.pdf]

Supplemental online material

Part 1 – Depiction of full raw sets of Raman spectra obtained for GRIS and IMC measured at q^{+} *= 4.55 °C·min*$^{-1}$*.*

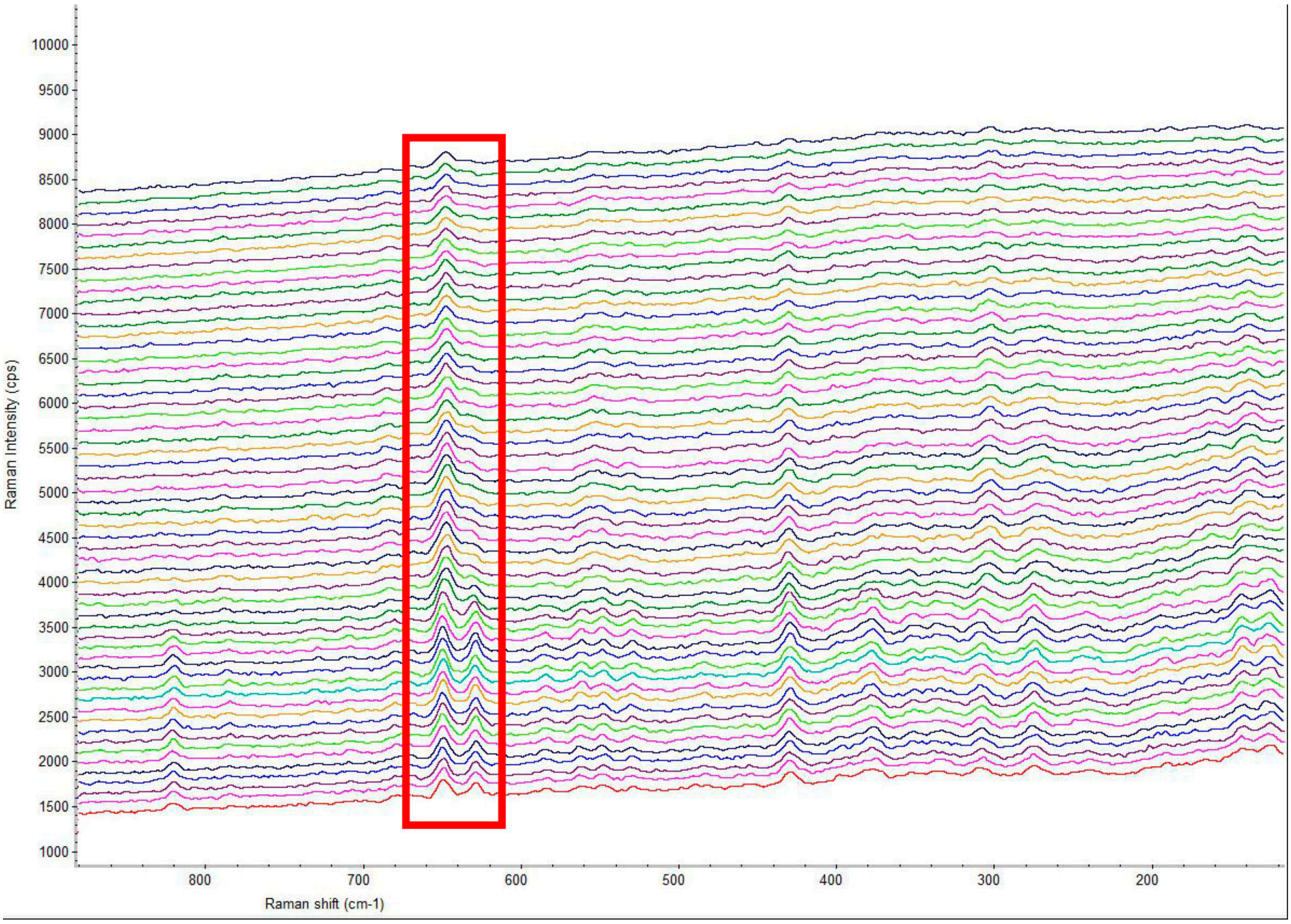

Figure S1: Example series of Raman spectra monitoring the crystallization of the 20 – 50 µm GRIS powder at q^{+} = 4.55 °C·min^{-1}. The framed part indicates the spectral region used to evaluate the degree of crystallinity by means of the multicomponent analysis.

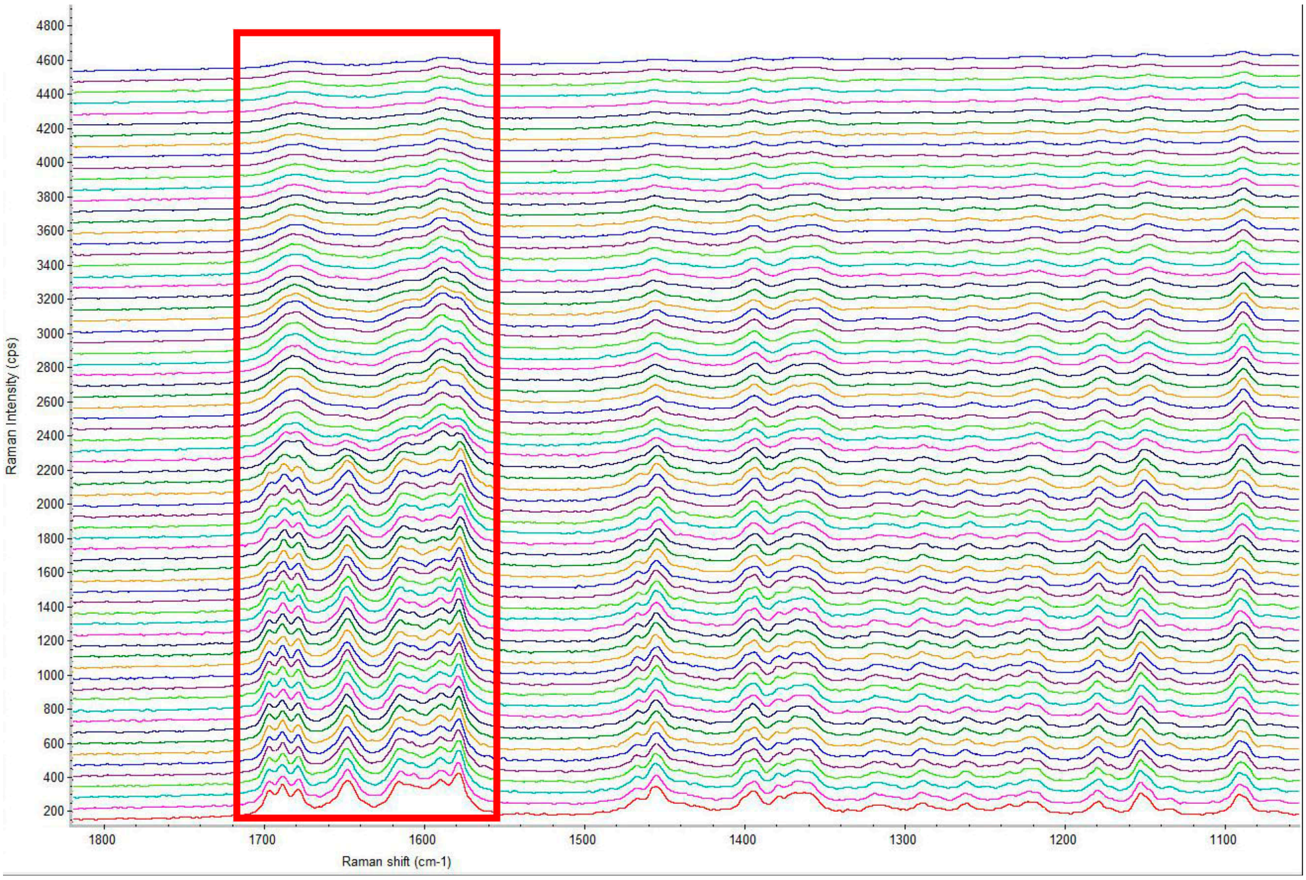

Figure S2: Example series of Raman spectra monitoring the crystallization of the 20 – 50 µm IMC powder at $q^{+} = 4.55$ °C·min^{-1}. The framed part indicates the spectral region used to evaluate the degree of crystallinity by means of the multicomponent analysis.

Part 2 – The results of the sc-MKA method applied to the DSC crystallization data of GRIS powder.

The reaction scheme for X independent reactions can be written as follows:

$$\frac{d\alpha_{c,x}}{dt} = A_x \cdot exp\left(-\frac{E_x}{RT}\right) \cdot \alpha_{c,x}^{M} \cdot \left(1-\alpha_{c,x}\right)^{N} \quad \text{(S1)}$$

$$1 = \sum_{x=1}^{n} \alpha_{c,x} \quad \text{(S2)}$$

where "x" is the index of the given individual sub-process, and "n" is the number of sub-processes (3 or 4 in the present case).

Table S1. Results of the sc-MKA method applied to the DSC data of the amorphous GRIS powder.

q^+	0.13	0.32	0.65	1.3	1.95	2.6	3.25	4.55	6.5	9.75
$logA_1$	15.21436	15.04244	14.9648	14.81066	14.46589	14.51564	14.43128	14.42654	14.26227	14.21229
E_1	110.4	110.4	110.4	110.4	110.4	110.4	110.4	110.4	110.4	110.4
N_1	0.080562	0.10484	0.37374	0.65799	0.78525	0.82883	1.08812	2.81207	2.48949	0.86195
M_1	1.10555	1.10018	1.0949	0.99541	0.79143	0.72643	0.71179	0.67113	0.59693	0.50794
$logA_2$	9.42583	9.34407	9.24087	9.05375	8.85183	8.93993	8.85001	8.94494	8.90569	9.05431
E_2	71.75	71.75	71.75	71.75	71.75	71.75	71.75	71.75	71.75	71.75
N_2	0.51007	0.44552	0.56805	0.71211	0.64032	0.6221	0.66662	0.56953	0.58968	0.68883
M_2	1.26693	1.25722	1.24873	1.19503	1.14246	1.15799	1.1459	1.13533	1.12695	1.13354
$logA_3$	8.27841	8.29484	8.13714	8.35301	8.24458	8.2137	8.12214	8.28077	8.38865	8.5008
E_3	71.75	71.75	71.75	71.75	71.75	71.75	71.75	71.75	71.75	71.75
N_3	0.48097	0.44282	0.44154	0.374	0.34665	0.31728	0.38964	0.39851	0.48201	0.57313
M_3	0.67996	0.72978	0.65756	0.83612	0.79394	0.78717	0.74573	0.793	0.87488	0.85869
$logA_4$	14.58194	14.41487	14.29546	14.31562						
E_4	110.4	110.4	110.4	110.4						
N_4	0.17239	0.18292	0.34669	0.62809						
M_4	0.77285	0.76416	0.7139	0.65727						
$\Delta H_1/\Delta H$	0.34519	0.22206	0.17744	0.20867	0.39421	0.22345	0.22838	0.27125	0.19338	0.048323
$\Delta H_2/\Delta H$	0.010269	0.11709	0.095194	0.2294	0.38297	0.42316	0.40237	0.35005	0.27356	0.36707
$\Delta H_3/\Delta H$	0.29422	0.2889	0.23164	0.17251	0.22282	0.35339	0.36925	0.3787	0.53306	0.584607
$\Delta H_4/\Delta H$	0.350321	0.37195	0.495726	0.38942						
ΔH	44.69939	52.56542	52.44967	49.00806	45.29159	42.22588	47.21337	60.71521	53.73503	58.80139
r	0.995282	0.995754	0.998134	0.998689	0.998201	0.999335	0.999286	0.999432	0.999702	0.999912

Part 3 – additional DSC data for amorphous indomethacin powder.

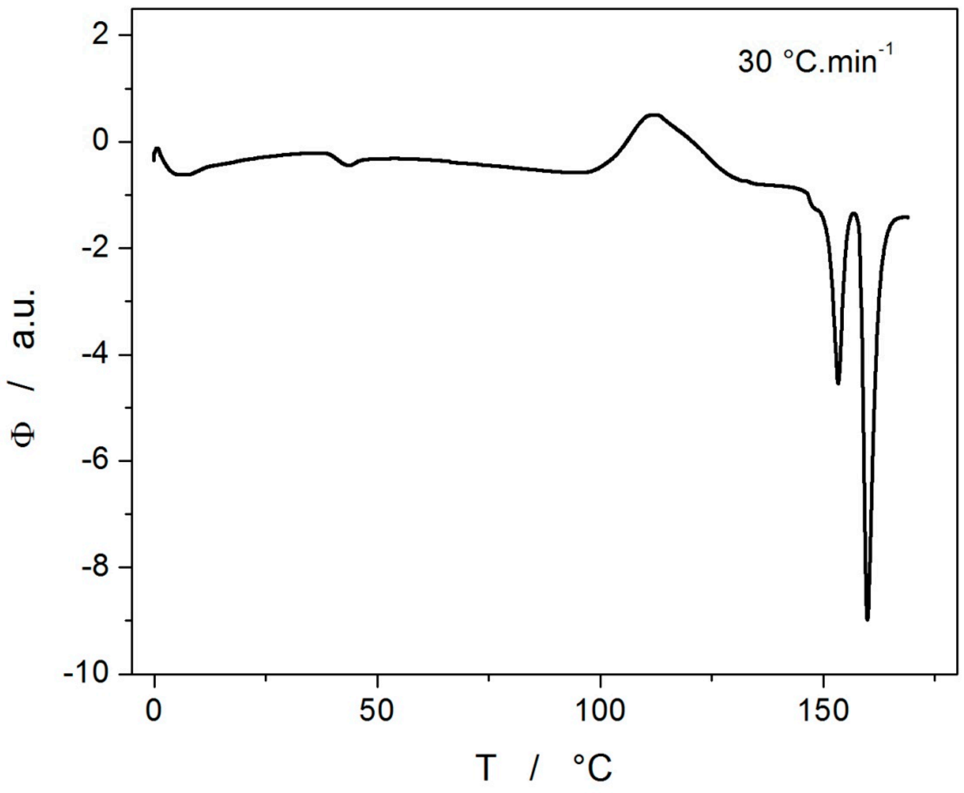

Figure S3. DSC curve obtained for amorphous indomethacin powder at q^{+} = 19.5 °C·min^{-1}
